# 

**Published:** 1885-12

**Authors:** 


### LIST OF SUBSCRIBERS

TO

### The Bristol Medico-Chirurgical Journal.

---

The attention of Subscribers is specially called to this list, in the hope that they will induce medical men who are not taking the *Journal* to support it both by subscription and by writing for it.

Names of intending Subscribers should be sent to the Assistant-Editor, L. M. Griffiths, 9 Gordon Road, Clifton, Bristol.

The Annual Subscription (if paid in advance) is 5/-, post free.

### England.

### CHESHIRE.

MACCLESFIELD.
T. S. Sheldon

### CORNWALL.

CALSTOCK.
H. T. Woodd

LISKEARD.
J. H. Jenkins

LOSTWITHIEL.
J. H. Sewart

MEVAGISSEY.
W. A. G. Laing

NEWQUAY.
T. Boyle

PADSTOW.
H. F. Marley

PENZANCE.
W. S. Bennett

POLRUAN.
W. H. Torbock

REDRUTH.
J. S. Hichens
G. A. Michell

ST. AUSTELL.
W. Mason

SALTASH.
H. B. Runnalls

### DEVONSHIRE.

ASHBURTON.
J. Adams

ASHTON.
S. Thomson

BAMPTON.
T. A. Guinness

BARNSTAPLE.
C. H. Gamble
J. Harper

BIDEFORD.
W. H. Ackland
J. Thompson

BLACK TORRINGTON.
H. H. Parsloe

BRIXHAM.
G. C. Searle

BROADCLYST.
J. Somer

CHAGFORD.
A. D. Hunt

CHILLINGTON.
F. H. Clarke

CHUDLEIGH.
J. A. Watson

COLLUMPTON.
S. R. Potter

DAWLISH.
A. de W. Baker
F. M. Cann

DEVONPORT.
G. T. Rolston
J. R. Rolston

DOLTON.
S. Michell

EXETER.
J. M. Ackland
A. W. Kempe
W. A. Moynan
S. R. Philipps
L. Shapter
E. B. Steele-Perkins
L. H. Tosswill

KINGSBRIDGE.
W. H. Webb

LYNTON.
F. C. Berry

MORCHARD BISHOP
W. J. C. Nourse

NEWTON ABBOT.
E. Haydon
W. G. Scott

PAIGNTON.
J. Alexander

PLYMOUTH.
R. H. Clay
Medical Society

ST. MARY CHURCH.
T. Finch

SALCOMBE.
A. H. Twining

SOUTH MOLTON.
E. Furse

TEIGNMOUTH.
J. A. Magrath
C. J. Workman

TIVERTON.
J. S. Smith

TORQUAY.
W. Powell

UFFCULME.
R. Bryden

WINKLEIGH.
J. H. Norman

### DORSETSHIRE.

SHERBORNE.
W. H. Williams

WIMBORNE.
A. J. H. Crespi

### ESSEX.

SOUTHEND.
E. E. Phillips

### GLOUCESTERSHIRE.

AVONMOUTH.
J. C. Heaven

BRISTOL.
G. Adams
J. B. Adams
O. Andrews
G. F. Atchley
T. G. L. Baretti
B. J. Baron
W. Basset
J. Beddoe
A. E. Blacker
A. F. Blagg
E. C. Board
J. Broom
A. Broster
G. F. Burder
J. P. Bush
A. Carr
W. F. Carter
J. P. Challacombe
J. M. Clarke
E. W. Coathupe
R. W. Coe
T. V. Coker
T. J. Colman
G. G. Corbould
F. R. Cross
A. G. Cunningham
J. Dacre
J. G. Davey
D. S. Davies
H. R. Dew
N. C. Dobson
C. H. Dowson
W. Dowson
E. L. W. Dunbar
T. F. Edgeworth
C. Elliott
J. F. Elwin
J. F. Evans
J. Ewens
R. G. Fendick
E. L. Fox
W. J. Fyffe
G. Gardiner
A. N. G. Gibbs
G. F. Giles
G. A. Gloag
C. W. Goodden
W. G. Grace
L. M. Griffiths
G. W. Grote
C. D. G. Hailes
A. J. Harrison
W. H. Harsant
S. Harwood
C. F. Hawkins
A. G. Hayman
C. K. C. Herapath
H. E. Hetling
F. F. Hills
Hospital, General
G. A. Imlay
Infirmary, Royal
G. W. Isaac
W. P. Keall
F. P. Lansdown
A. E. A. Lawrence
H. Lawrence
H. Laxton
J. R. Lewis
Library, Museum and
W. E. Lloyd
F. T. B. Logan
W. C. Luffman
W. C. Lysaght
H. Marshall
C. E. Matthews
T. O. Mayor
J. Milne
C. Newman
J. Newstead
T. D. Nicholson
J. A. Norton
T. C. Norton
J. H. Parry
T. C. Parson
J. D. F. Parsons
G. C. P. Pauli
W. J. Penny
J. H. Perkins
W. H. Phelps
C. F. Pickering
W. E. Pountney
A. Prichard
A. W. Prichard
J. E. Prichard
A. Pring
A. B. Prowse
G. Rogers
W. B. Roué
C. K. Rudge
H. T. Rudge
J. E. Shaw
E. J. Sheppard
W. E. Shorland
E. M. Skerritt
G. M. Smith
J. G. Smith
R. S. Smith
S. Smith
W. H. Spencer
G. M. Stansfeld
C. Steele
L. E. W. Stephens
G. Stock
F. W. Stoddart
J. G. Swayne
S. H. Swayne
F. Taylor
J. Taylor
G. S. Thomson
H. C. Thurston
W. J. Tivy
H. Waldo
C. H. Wallace
J. H. Wathen
T. Webster
M. Willett

CHELTENHAM.
J. Bubb
J. C. Gooding
Library, Cheltenham
C. J. Newton
L. Winterbotham

CHIPPING SODBURY.
A. Grace

CIRENCESTER.
O. H. Fowler

DOWNEND.
H. Skelton

DURSLEY.
F. J. Joynes

FAIRFORD.
J. Cornwall

FISHPONDS.
H. R. Mead

FRAMPTON-ON-SEVERN.
T. Watts

GLOUCESTER.
R. W. Batten
F. T. Bond
E. D. Bower
A. M. Sydney-Turner
H. H. Tomkins
J. P. Wilton

HAMBROOK.
E. Crossman
W. F. B. Eadon

KINGSWOOD.
H. Grace

LYDBROOK.
H. S. Fletcher

LYDNEY.
A. S. Currie

MARSHFIELD.
J. Reader

NORTHWOODS.
R. Eager

### GLOUCESTERSHIRE *(Continued).*

STAPLE HILL.
W. Murray

STAPLETON.
G. Thompson

STOKE BISHOP.
J. Stewart
D. H. Walsh
F. F. Welsh

STONEHOUSE.
G. T. B. Watters

STOW-ON-THE-WOLD.
E. Dening

STROUD.
A. S. Cooke
E. A. Howsin
W. H. Paine
F. W. Storry

TETBURY.
W. Wickham

THORNBURY.
E. M. Grace
W. G. Salmon
T. H. Taylor

WESTBURY-ON-TRYM.
H. Ormerod
G. Smith

WINCHCOMBE.
C. Penruddocke

WOTTON-UNDER-EDGE
D. H. Forty

### HAMPSHIRE.

BOURNEMOUTH.
D. C. Embleton
V. Fasulo
E. P. Philpots

### HEREFORDSHIRE.

HEREFORD.
W. C. Whitfeld

ROSS.
J. W. Norman

### LANCASHIRE.

LIVERPOOL.
Medical Institution.

MANCHESTER.
J. Scott

POULTON-LE-FYLDE.
P. H. Day

### MIDDLESEX.

ENFIELD.
W. B. Kesteven

LONDON.
H. Cooper
G. S. Gregory
C. M. Jessop
W. J. Seward
Society, Royal Medical and Chirurgical

### MONMOUTHSHIRE.

ABERGAVENNY.
S. H. Steel

CAERLEON.
J. A. Morris

CHEPSTOW.
A. G. Lawrence

EBBW VALE.
J. W. Davies
J. Sheehy

MONMOUTH.
G. Wilson

NEWPORT.
H. R. Hudson
O. E. B. Marsh
A. G. Thomas

PONTYPOOL.
S. B. Mason

RHYMNEY.
T. H. Redwood

RISCA.
G. B. Robathan

TINTERN.
T. W. A. Napier

TREDEGAR.
G. A. Brown

### NORTHAMPTONSHIRE.

KETTERING.
R. E. Burges

### OXFORDSHIRE.

OXFORD.
R. W. Doyne

### SOMERSETSHIRE.

AXBRIDGE.
G. Smith

BANWELL.
A. J. Bisdée

BATH.
W. B. Beatson
F. H. Blaxall
E. J. Cave
T. Cole
F. S. Cowan
A. E. W. Fox
H. W. Freeman
H. F. A. Goodridge
T. B. Goss
F. K. Green
H. C. Hopkins
L. J. King
T. P. Lowe
F. Mason
M. W. H. Russell
R. J. H. Scott
E. Skeate
J. K. Spender
H. G. Terry

BRIDGWATER.
F. J. C. Parsons
J. B. Sincock

BRISLINGTON.
B. B. Fox
C. H. Fox

BRUTON.
F. Stockwell

BURNHAM.
D. A. Fraser

### SOMERSETSHIRE *(Continued).*

CLEVEDON.
T. Davis
G. F. P. Pizey
S. Skinner

COMPTON MARTIN.
W. F. Lovell

FROME.
E. F. H. Burroughs
F. Parsons
F. E. Pearse

ILMINSTER.
S. D. Hine
C. Munden

KEYNSHAM.
N. Crisp
J. Lodge
R. W. Thomas

LANGPORT.
J. Morgan

LONG ASHTON.
J. Fuller

MIDSOMER NORTON.
E. Blacker
A. Waugh

MILVERTON.
C. Randolph

MINEHEAD.
T. Clark
T. J. Ollerhead

NORTH PETHERTON.
W. J. Todd

PILL.
A. H. Boys

PORTISHEAD.
L. A. Weatherly

SHEPTON MALLET.
B. N. Hyatt

SOMERTON.
E. W. Valentine

SOUTH PETHERTON.
W. H. Walter

TAUNTON.
H. J. Alford
J. Carey
W. Liddon

TEMPLE CLOUD.
T. Martin

WEDMORE.
R. P. Tyley

WELLINGTON.
J. Meredith
T. E. P. Prideaux

WELLS.
W. Fairbanks
A. L. Wade

WESTON-SUPER-MARE.
G. E. Alford
G. B. Fraser
E. F. Martin
W. H. G. Phelps
G. F. Rossiter
R. Roxburgh
F. W. S. Wicksteed

WINCANTON.
J. B. Colthurst

WRINGTON.
H. W. Collins

YATTON.
A. de C. Lyons

YEOVIL.
P. S. H. Colmer
C. J. Marsh

### WILTSHIRE.

BRADFORD-ON-AVON.
W. D. Lovell

CHIPPENHAM.
G. H. Daly

DEVIZES.
T. B. Anstie
C. Eddowes

LACOCK.
J. H. Crisp

MALMESBURY.
R. Kinneir

SALISBURY.
B. C. Gowing
H. J. Manning
T. Sanctuary

STRATTON ST. MARGARET.
J. Fernie

SWINDON.
J. B. Fry
W. Howse
J. C. Maclean

TROWBRIDGE.
G. C. Tayler
N. V. Wise

WARMINSTER.
J. Hinton
R. L. Willcox

WOOTTON BASSETT.
J. M. Kirkman

### Ireland.

### ANTRIM.

BELFAST.
J. W. Browne

### Scotland.

### EDINBURGHSHIRE.

EDINBURGH.
Y. J. Pentland

### Wales.

### BRECONSHIRE.

BRECON.
D. V. Rees

SENNYBRIDGE.
W. R. Jones

TALGARTH.
W. Howells

### CARDIGANSHIRE.

LAMPETER.
E. C. Davies

NEW QUAY.
E. R. Jones

### CARMARTHENSHIRE.

CARMARTHEN.
G. J. Hearder

FERRYSIDE.
P. Williams

LLANGENNECH.
A. Devonald

### GLAMORGANSHIRE.

ABERAMAN.
J. B. James

ABERDARE.
E. Jones

CAERPHILLY.
J. Lewellyn

CARDIFF.
A. P. Fiddian
Medical Society
J. Milward
W. Morgan
W. Price
A. Sheen
C. T. Vachell

MERTHYR TYDVIL.
J. L. W. Ward

SWANSEA.
J. Couch
J. F. Fry
G. Padle
J. A. Rawlings
J. Thomas

### INDEX.

Aachen—Dr. E. P. Philpots, 104.
Abdominal tumour (*Extract*), 137.
Abdominal tumours, On—Sir S. Wells (*Review*), 132.
Addison's disease—J. E. Prichard, 269.
American Surgical Association, Transactions of (*Review*), 133.
Antipyrine (*Extracts*), 142, 206.
Aorta, Aspiration of—J. Dacre, 189.
Aorta, Rupture of—J. P. Bush, 129; Dr. H. Marshall, 178; A. Prichard, 127.

Barnes, Drs. R. and F.—A system of obstetric medicine and surgery (*Review*), 279.
Behrens, Dr. W. J.—Text-book of botany (*Review*), 135.
Boracic acid, Action and use of (*Extract*), 70.
Botany, Text-book of—Dr. W. J. Behrens (*Review*), 135.
Brain of lioness, Morbid appearances in—Dr. W. B. Kesteven, 246.
Bush, J. P.—Rupture of aorta, 129.

Cancer, Parasitic origin of (*Extract*), 142.
Cholera (*Extract*), 286.
Chorea (*Extract*), 207.
Chudleigh, Rev. R. A.—A method of expressing ear-power, 159.
Churchill, F.—Face and foot deformities (*Review*), 203.
Clarke, W. M.—Intestinal obstruction, 38; obituary notice of, 287.
Cocaine, Uses of (*Extracts*), 61, 284.
Corn cures (*Extract*), 70.
Cotterell, E.—Common injuries to limbs (*Review*), 135.
Creighton, Dr. C.—Unconscious memory in disease (*Review*), 281.
Cremation—Dr. J. G. Davey, 81.
Cross, F. R.—Intestinal obstruction, 34.

Dacre, J.—Aspiration of aorta, 189.
Davey, Dr. J. G.—Cremation, 81.
Deakin, S.—Pendulous fatty tumour of thigh, 51.
Deformities, Bodily—H. A. Reeves (*Review*), 200.
Diabetes, Treatment of (*Extract*), 67.
Diathylacetal (*Extract*), 63.
Diphtheria—Dr. A. Sheen, 113.
Diphtheria, Tracheotomy in laryngeal—R. W. Parker (*Review*), 199.
Dobson, N. C.—Intestinal obstruction, 28.

Ear-power, A method of expressing—Rev. R. A. Chudleigh, 159.
Ebstein, Dr. W.—The regimen in gout (*Review*), 131.
Elliott, Dr. C.—A case of hyperpyrexia, 109.
Erysipelas and the antiseptic method (*Extract*), 136.
Evans, Dr. J. F.—A unique case of attempted suicide, 124.
Explosive drugs (*Extract*), 69.
Eye, Diseases of the—H. R. Swanzy (*Review*), 58.

Face and foot deformities—F. Churchill (*Review*), 203.
Facial neuralgia—H. B. Runnalls, 249.
Fæces, Retention and accumulation of—Dr. J. E. Shaw, 43.
Femur, Fracture of—C. F. Pickering, 48.
Fingers, Wounds of—Dr. J. K. Spender, 73.
Fox, Dr. E. Long—Enlargement of spleen, 145; intestinal obstruction, 17; Mangana water, 47.

Gaultheriæ, Oleum (*Extract*), 63.
Goître, Parenchymatous (*Extract*), 141.
Goodridge, Dr. H. F. A.—Intestinal obstruction, 40.
Gould, A. P.—Elements of surgical diagnosis (*Review*), 58.
Gout and its relations to diseases of liver and kidneys—Dr. R. Roose (*Review*), 197.
Gout in its clinical aspects—Dr. J. M. Granville (*Review*), 197.
Gout, The regimen in—Dr. W. Ebstein (*Review*), 131.

Granville, Dr. J. M.—Gout in its clinical aspects (*Review*), 197.

Hare, Drs. G. S. Woodhead and A. W.—Pathological mycology (*Review*), 202.
Hip, Excision of (*Extract*), 213.
Histological specimens, Preparation of (*Extract*), 212.
Hyperpyrexia, A case of—Dr. C. Elliott, 109.

Incompatibility of drugs (*Extract*), 215.
Infants, Management of new-born (*Extract*), 66.
Insanity, The border-land of—Dr. W. B. Kesteven, 97.
Intestinal obstruction—W. M. Clarke, 38; F. R. Cross, 34; N. C. Dobson, 28; Dr. E. Long Fox, 17; Dr. H. F. A. Goodridge, 40; Dr. E. M. Skerritt, 36; J. Greig Smith, 1; Dr. R. Shingleton Smith, 20; F. Treves, 13, (*Review*), 60.
Intestinal polypus—H. B. Runnalls, 181.
Iodoform, Fatal poisoning by (*Extract*), 210.

Jaccoud, Prof. S.—The curability and treatment of pulmonary phthisis (*Review*), 52.
James, Dr. P.—A guide to the new Pharmacopœia (*Review*), 277.

Keetley, C. B.—An index of surgery (*Review*), 278.
Kesteven, Dr. W. B.—Morbid appearances in brain of lioness, 246; the border-land of insanity, 97.
Kidneys, Diseases of the—C. H. Ralfe (*Review*), 281.

Lead-poisoning, A new symptom of (*Extract*), 71.
Leamington, The saline waters of—Dr. F. W. Smith (*Review*), 280.
Limbs, Common injuries to—E. Cotterell (*Review*), 135.
Locomotor ataxia and syphilis (*Extract*), 65.
Lupus and tuberculosis (*Extract*), 211.
Lymphadenosis—Dr. J. Meredith, 255.

Mangana water—Dr. E. Long Fox, 47.
Marshall, Dr. H.—Rupture of aorta, 178.
Materia medica and pharmacy, Notes on—Dr. F. T. Roberts (*Review*), 57.
Maunsell, S. E.—Medical experiences in India (*Review*), 280.
Medical experiences in India—S. E. Maunsell (*Review*), 280.
Memory in disease, Unconscious—Dr. C. Creighton (*Review*), 281.
Mental disease, Lectures on—Dr. W. H. O. Sankey (*Review*), 56.
Meredith, Dr. J.—Lymphadenosis, 255.

Neuropathic sneezing (*Extract*), 216.
Night-sweats, External treatment of (*Extract*), 215.

Obesity, Treatment of (*Extract*), 285.
Obstetric medicine and surgery, A system of—Drs. R. and F. Barnes (*Review*), 279.
Ostitis of the hip, Chronic (*Extract*), 67.
Otology, Corrosive sublimate in (*Extract*), 139.

Paraldehyde (*Extract*), 143.
Paralysis of oculo-motor nerve, Periodically recurring (*Extract*), 62.
Parker, R. W.—Tracheotomy in laryngeal diphtheria (*Review*), 199.
Parry, J. S.—Extra-uterine pregnancy (*Review*), 135.
Pathology, Contributions to—Dr. J. R. Wardell (*Review*), 278.
Pathological mycology—Drs. G. S. Woodhead and A. W. Hare (*Review*), 202.
Pathology, Practical—Dr. G. S. Woodhead (*Review*), 60.
Pendulous fatty tumour of thigh—S. Deakin, 51.
Pepper, A. J.—Surgical pathology (*Review*), 205.
Pepsin in gastric juice, Amount of (*Extract*), 140.
Peptonoids, Carnrick's beef (*Extract*), 64.
Perspiration, Agaricine in (*Extract*), 216.

Pharmacopœia, A guide to the new —Dr. P. James (*Review*), 277.
Pharmacopœia, The British, 1885 (*Review*), 274.
Philpots, Dr. E. P.—Aachen, 104.
Phthisis, The curability and treatment of pulmonary — Prof. S. Jaccoud (*Review*), 52.
Pickering, C. F.—Fracture of femur, 48.
Pregnancy, Extra-uterine — J. S. Parry (*Review*), 135.
Prichard, A.—Rupture of aorta, 127.
Prichard, J. E.—Addison's disease, 269.
Pulmonary (Intra-) injections—Dr. R. Shingleton Smith, 164.
Pustule, Malignant (*Extract*), 64.
Pyrexia—Dr. W. H. Spencer, 217.

Ralfe, C. H.—Diseases of the kidneys (*Review*), 281.
Reeves, H. A.—Bodily deformities (*Review*), 200.
Rheumatism, Acute articular (*Extract*), 137.
Rhinolith, Impacted (*Extract*), 283.
Ring finger in musicians, Liberating the (*Extract*), 65.
Roberts, Dr. F. T.—Notes on materia medica and pharmacy (*Review*), 57.
Roberts, J. B.—Surgical delusions and follies (*Review*), 206.
Roose, Dr. R.—Gout and its relations to diseases of liver and kidneys (*Review*), 197.
Runnalls, H. B.—Facial neuralgia, 249; intestinal polypus, 181.

Sankey, Dr. W. H. O.—Lectures on mental disease (*Review*), 56.
Shaw, Dr. J. E.—Retention and accumulation of fæces, 43.
Sheen, Dr. A.—Diphtheria, 113.
Skerritt, Dr. E. M. — Intestinal obstruction, 36.
Smith, Dr. F. W.—The saline waters of Leamington (*Review*), 280
Smith, Dr. R. Shingleton — Intestinal obstruction, 20; intra-pulmonary injections, 164.
Smith, J. Greig—Intestinal obstruction, 1.
Spencer, Dr. W. H.—Pyrexia, 217.
Spender, Dr. J. K. — Wounds of fingers, 73.
Spinal caries—H. H. Tomkins, 184.
Spleen, Enlargement of — Dr. E. Long Fox, 145.
Splenic fever (*Extract*), 141.
Suicide—W. W. Westcott (*Review*), 204.
Suicide, A unique case of attempted —Dr. J. F. Evans, 124.
Surgery, An index of—C. B. Keetley (*Review*), 278.
Surgical delusions and follies—J. B. Roberts (*Review*), 206.
Surgical diagnosis, Elements of — A. P. Gould (*Review*), 58.
Surgical pathology — A. J. Pepper (*Review*), 205.
Swanzy, H. R.—Diseases of the eye (*Review*), 58.

Terpine (*Extract*), 284.
Thalline (*Extract*), 208.
Thomas, H. O.—What is recognised treatment? (*Review*), 201.
Thompson, Sir H. — Stricture of urethra (*Review*), 134.
Thomsen's disease (*Extracts*), 208, 209.
Time to take medicines, The proper (*Extract*), 214.
Tomkins, H. H.—Spinal caries, 184.
Treatment, What is recognised?— H. O. Thomas (*Review*), 201.
Treves, F.—Intestinal obstruction, 13, (*Review*), 60.
Tuberculosis in families, Pulmonary (*Extract*), 138.

Urethra, Stricture of — Sir H. Thompson (*Review*), 134.

Wardell, Dr. J. R.—Contributions to pathology, &c. (*Review*), 278.
Wells, Sir S. — On abdominal tumours (*Review*), 132; the revival of ovariotomy (*Review*), 132.
Westcott, W. W.—Suicide (*Review*), 205.
Whooping cough (*Extracts*), 283, 284.
Woodhead, Dr. G. S. — Practical pathology (*Review*), 60.
Woodhead and A. W. Hare, Drs. G. S. — Pathological mycology (*Review*), 202.

J. W. ARROWSMITH, PRINTER, QUAY STREET, BRISTOL.

### EDITORIAL NOTICE.

THE gratifying success of this Journal has stimulated us to try to improve it. The volume for 1886 will be marked by several new departures, which we trust will all be in the right direction. The Publication Secretary, Mr. GRIFFITHS, will help in Editorial work, and as Assistant Editor, will have charge of a small department which will be concerned with the social, literary, and anecdotal aspects of our profession. The department of "Medical Extracts" will be transformed into a chronicle of medical and surgical science and practice, in which translations, abstracts, and periscopes will appear, signed by the writers. The department of "Notes on Books" will be amplified and extended into Reviews, and occasionally, where the importance of the works seems to justify it, into summaries. "Original Papers" and "Clinical Records" will remain as at present.

